# Supplementary material for: Precision measurements on oxygen formation in stellar helium burning with gamma-ray beams and a Time Projection Chamber
Source: Nat Commun. 2021 Oct 11;12:5920. doi: 10.1038/s41467-021-26179-x (PMC8505630; doi:10.1038/s41467-021-26179-x)
Supplement: Supplementary file 2 — Description of Additional Supplementary Files [file 41467_2021_26179_MOESM2_ESM.pdf]

Description of Additional Supplementary Files

Supplementary Data 1: This Microsoft Excel file contains data pertaining to the experimental measurements presented in the manuscript. The first tab contains the measured total cross sections at each of the effective centre-of-mass energies. The second tab contains the centre-of-mass angles, θ, that were measured for each event at each of the effective centre-of-mass energies. The third tab contains the angular efficiencies at each of the effective centre-of-mass energies, which permit the partial wave decomposition fits to the experimental data. The fourth tab contains the best fit parameters of the partial wave decomposition to each of the angular distributions, along with their 1σ errors. Finally, the fifth tab contains a table describing the systematic errors and their sources.
